# APRIL Induces a Novel Subset of IgA^+^ Regulatory B Cells That Suppress Inflammation via Expression of IL-10 and PD-L1

**DOI:** 10.3389/fimmu.2019.01368

**Published:** 2019-06-14

**Authors:** Cynthia M. Fehres, Nathalie O. van Uden, Nataliya G. Yeremenko, Leticia Fernandez, Gabriela Franco Salinas, Leonie M. van Duivenvoorde, Bertrand Huard, Jacques Morel, Hergen Spits, Michael Hahne, Dominique L. P. Baeten

**Affiliations:** ^1^Department of Clinical Immunology and Rheumatology, Amsterdam Rheumatology and Immunology Centre, Amsterdam UMC, University of Amsterdam, Amsterdam, Netherlands; ^2^Department of Experimental Immunology, Amsterdam UMC, University of Amsterdam, Amsterdam, Netherlands; ^3^Centre National de la Recherche Scientifique, Universite de Montpellier, Montpellier, France; ^4^Institute for Advanced Biosciences, INSERM U1209, University Grenoble Alpes, Grenoble, France; ^5^Department of Rheumatology, CHU de Montpellier, Montpellier University, Montpellier, France; ^6^Amsterdam UMC, University of Amsterdam, and AIMM Therapeutics, Amsterdam, Netherlands

**Keywords:** APRIL, Immunoregulation, IgA, IL-10, inflammation, regulatory B cells

## Abstract

Regulatory B cells (Bregs) are immunosuppressive cells that modulate immune responses through multiple mechanisms. The signals required for the differentiation and activation of these cells remain still poorly understood. We have already shown that overexpression of A PRoliferation-Inducing Ligand (APRIL) reduces the incidence and severity of collagen-induced arthritis (CIA) in mice. Furthermore, we have described that APRIL, but not BAFF, promoted IL-10 production and regulatory functions in human B cells. Therefore, we hypothesized that APRIL, but not BAFF, may be involved in the induction and/or activation of IL-10 producing Bregs that suppress inflammatory responses *in vitro* and *in vivo*. Here, we describe that APRIL promotes the differentiation of naïve human B cells to IL-10-producing IgA^+^ B cells. These APRIL-induced IgA^+^ B cells display a Breg phenotype and inhibit T cell and macrophage responses through IL-10 and PD-L1. Moreover, APRIL-induced IL-10 producing Bregs suppress inflammation *in vivo* in experimental autoimmune encephalitis (EAE) and contact hypersensitivity (CHS) models. Finally, we showed a strong correlation between APRIL and IL-10 in the inflamed synovial tissue of inflammatory arthritis patients. Collectively, these observations indicate the potential relevance of this novel APRIL-induced IgA^+^ Breg population for immune homeostasis and immunopathology.

## Introduction

Over the past decade, Bregs have emerged as a novel subset of cells implicated in the inhibition of excessive inflammation. Early studies showed that Bregs suppress inflammation by the production of IL-10 in models of colitis, EAE and arthritis (1–3). Through the expression of various immune-regulatory molecules, Bregs suppress Th1 and Th17 differentiation, skew T cell differentiation in favor of a regulatory phenotype and dampen activation of myeloid cells [reviewed in (4)]. Bregs have been reported to be functionally and numerically impaired in several autoimmune diseases (5, 6) as well as in chronic graft-vs.-host disease (7, 8) and cancer (9). As such, maintaining and/or enhancing Breg number and function is of potential therapeutic interest to control immune-mediated inflammatory diseases.

In contrast to the well-described regulatory functions of Bregs, the phenotypical and molecular identity of these cells is still not fully elucidated. In mice, IL-10 producing Bregs comprise subsets expressing CD1d, such as CD1d^hi^CD5^−^B220^low^CD11b^+^IgM^+^ (3), CD1d^h^iCD5^+^ (10), and CD1d^hi^TIM^+^CD5^+^ (11) B cells. In addition, peritoneal CD5^+^ B1 cells have been described as potent IL-10 producing Bregs, as well as CD21^hi^CD23^hi^CD24^hi^ transitional type 2 marginal zone precursors (12), CD138^+^ plasmablasts (13), and IgA^+^ plasmocytes (14, 15). Although less is known about human Bregs, they have been reported within subsets of CD19^+^CD24^hi^CD38^hi^CD1d^+^ transitional B cells (6), CD24^hi^CD27^+^ B cells (16), and CD24^−^CD27^hi^CD38^hi^ plasmablasts (7). The phenotypical heterogeneity of Breg subsets and the inability to identify a Breg lineage-specific transcription factor or marker in both mice and human, lead to the hypothesis that Bregs do not represent a distinct cellular lineage but might potentially differentiate from any B cell in response to appropriate environmental stimuli (4). However, the signals required for this differentiation and activation of murine and/or human Bregs remain still poorly understood.

A Proliferation Inducing Ligand (APRIL) and B cell Activating Factor (BAFF) are two members of the TNF family known to modulate B cell responses. BAFF and APRIL share binding to two receptors: transmembrane activation and calcium-modulating cyclophilin-ligand interactor (TACI) and B cell maturation antigen (BCMA) (17, 18). In addition, BAFF binds specifically to the BAFF-receptor, while APRIL can also bind to heparan sulfate proteoglycans (19, 20). BAFF overexpression in the mouse induces multi-organ autoimmune disease, including systemic lupus erythematosus (SLE) and IgA nephritis like disease manifestations characterized by increased anti-ds-DNA and anti-nuclear autoantibody production, renal Ig complex deposition, and raised serum IgA, IgG, and IgE levels (21, 22). In patients with SLE, elevated levels of BAFF are found in the serum, which correlates with raised anti-ds-DNA autoantibody levels (23). Therapeutic blockade of BAFF using the monoclonal antibody belimumab has been approved for treatment of SLE (24, 25). In contrast to BAFF, APRIL transgenic (Tg) mice do not develop spontaneous autoimmune disease and, on the contrary, are even protected against collagen-induced arthritis, which was partly mediated by a decreased production of collagen-specific autoantibodies (26). More recently, we have described that APRIL, but not BAFF, promoted IL-10 production and regulatory functions in human B cells (27).

Based on these observations, we hypothesized that APRIL, but not BAFF, may be involved in the induction and/or activation of IL-10 producing Bregs that suppress inflammatory responses. We therefore investigate the effects of APRIL on the development and/or activation of human Bregs and characterized the phenotype, functions and molecular regulation of APRIL-induced regulatory B cells *in vitro* and *in vivo*.

## Materials and Methods

### Mice

The generation of APRIL-Tg mouse has been described (28). In all experiments performed littermates were used as control mice.

EAE was induced by subcutaneous immunization with 75 μg MOG peptide (aminoacids 35-55; GenScript Co.) in CFA (Chondrex) at two different sites in the neck. Subsequently, 300 ng of pertussis toxin was injected in two portions of 150 ng on the same day and 2 days after MOG immunization. The development of EAE and the weight of the animals was monitored daily for 30 days. Neurological signs of disease will be scored using a five-point scale (0: normal, 1: flaccid tail, 2: partial hind limb paresis/paralysis, 3: complete hind limb paralysis, 4: front limb paralysis, 5: moribund). At the end of the experiment, the incidence and severity of EAE were calculated per mouse cohort.

Peritoneal B cells were depleted, as previously described (29). Mice received intraperitoneal injections of distilled water (0.5 ml) twice a week 3 weeks prior to induction of EAE. Control mice were injected with 0.5 ml PBS. Depletion of peritoneal B1 cells after injection with distilled water was confirmed by FACS analysis.

Animal experiments were performed in compliance with national and institutional guidelines. Experiments were authorized by the respective French authorities (Départementale des service vétérinaires de la Prefecture de l'Herault), Permit number D 34-172-16.

### Antibodies and Flow Cytometry

Cells were stained at 4°C in PBS. Expression of IL-10 was analyzed using the PE-labeled IL-10 secretion assay according to manufacturer's instructions (Miltenyi Biotec). The following antibodies were used: FITC-conjugated IgA (DAKO), FITC-conjugated FoxP3 (150D/E4, eBioscience), PE-conjugated IgD (IA6-2, BD), PE-conjugated IgG (Southern Biotech), PE-conjugated CD86 (2331, BD), Pe-CF594-conjugated PD-L1 (MIH1, BD), PE-Cy7-conjugated CD4 (SK3, BD), Pe-Cy7-conjugated CD40 (5C3, BioLegend), APC-conjugated CD27 (O323, eBioscience), APC-conjugated TNF (Mab11, eBioscience), AF-700-conjugated HLA-DR (LN3, eBioscience), and APC-Cy7-conjugated CD19 (SJ25C1, BD). Expression of IL-10 was analyzed using the PE-labeled IL-10 secretion assay (Miltenyi Biotec). For intracellular TNF staining, cells were fixed using the BD cytofix/Cytoperm kit (BD) according to manufacturer's instructions. For FoxP3, cells were fixed and permeabilized with FoxP3 staining buffer set (eBioscience) following the manufacturer's instructions. Proliferation of cells was assessed using brilliant violet 431-labeled cell trace (Molecular Probes) according to manufacturer's protocol. B cells were gated based on forward scatter (FSc) and side scatter (SSc) and CD19 positivity. Appropriate isotype controls were taken along in all experiments. Cells were analyzed on a FACSCanto II or LSR Fortessa (both BD) and data were analyzed with FlowJo software (TreeStar).

### Patient Material

Synovial fluids (SF) were obtained in the CHU Montpellier and the Academic Medical Center Amsterdam from 24 patients with inflammatory arthritis after written informed consent was received. SF was centrifuged to remove cells and treated with hyaluronidase (20 U/ml) for 30 min, 37°C to reduce the viscosity of the SF.

### Generation and Culture of Human B Cell Clones

Peripheral blood mononuclear cells (PBMCs) derived from healthy donors were isolated using ficoll-Paque. CD19^+^ B cells were subsequently MACS-isolated using CD19 beads (Miltenyi Biotec), where after bulk CD19^+^IgD^+^CD27^−^ naïve, CD19^+^IgM^+^CD27^+^ memory IgM, CD19^+^IgG^+^CD27^+^ memory IgG and CD19^+^IgA^+^CD27^+^ memory IgA B cells were FACS-sorted. These bulk B cells were transduced with a Bcl-6-Bcl-XL-containing construct by retrovirus-mediated gene transfer and clones of these cells were obtained followed by single cell sorting as previously reported (30). B cells clones are cultured in IMDM supplemented with 10% FCS, 100 IU/ml penicillin and 50 μg/ml streptomycin in the presence of irradiated CD40L-expressing fibroblasts and 50 ng/ml recombinant mouse IL-21.

### Culture of Primary Human Peripheral Blood-Derived Naïve B Cells

Cells are cultured in IMDM supplemented with 10% FCS, 100 IU/ml penicillin and 50 μg/ml streptomycin. PBMCs derived from healthy donors were isolated using ficoll-Paque. CD19^+^ B cells were subsequently MACS-isolated using CD19 beads (Miltenyi Biotec), where after CD19^+^IgD^+^CD27^−^ naïve B cells were FACS-sorted. 2 ×10^5^ Naïve B cells were cultured for 6 days on 50.000 irradiated CD40L-expressing fibroblasts in the presence of 50 ng/ml recombinant mouse IL-21, 0,5 ng/ml rhTGF-β (R&D Systems), 100 ng/ml rhAPRIL (R&D Systems) and 100 ng/ml rhBAFF (R&D Systems). When stated, blocking antibodies to TACI and BCMA (both 10 μg/ml, R&D systems) were added to the cultures. In some experiments, B cells were additionally stimulated for 2 days with 10 ng/ml LPS (Invivogen), 10 μg/ml CpG (ODN2006, Invivogen) or medium as control after the 6 day culture protocol.

### Co-culture of Autologous B Cells and CD4^+^ T Cells

Sorted naïve human B cells were isolated and cultured as described above. Autologous CD4^+^ T cells were FACS-sorted as CD4^+^ cells and frozen in liquid nitrogen for 6 days. After 6 days of culture of the B cells, cells that still expressed a naïve phenotype (CD19^+^IgD^+^CD27^−^) were FACS-sorted from recently class switched IgA^+^ B cells. The CD4^+^ T cells were thawed, stained with cell trace violet and stimulated with CD3 CD28 dynabeads (Gibco Life Technologies) according to manufacturer's instructions. Autologous sorted B cells and CD4^+^ T cells were co-cultured in round bottom 96 wells plates for 3–6 days in a 1:5 ratio. When indicated, neutralizing antibodies to PD-L1 (10 μg/ml, R&D Systems) and/or IL-10 (10 μg/ml, BD) were added.

### Co-culture of Autologous B and GM-CSF Macrophages

Sorted naïve human B cells were isolated and cultured as described above. Autologous CD14^+^ monocytes were isolated from the flow-through of the CD19 MACS using CD14 beads (Miltenyi Biotech). CD14^+^ monocytes were cultured for 6 days in the presence of 20 ng/ml GM-CSF (Invitrogen). Medium was replenished at day 3 of the culture. After 6 days of culture of the B cells, cells that still expressed a naïve phenotype (CD19^+^IgD^+^CD27^−^) were FACS-sorted from recently class switched IgA^+^ B cells. The macrophages were harvested from the wells using TrypLE select (ThermoFisher) and stimulated for 30 min, 37°C with LPS (100 ng/ml). Sorted B cells and macrophages were co-cultured in round bottom 96 wells plates for 3 additional days in a 1:4 ratio. When indicated, neutralizing antibodies to PD-L1 (10 μg/ml, R&D Systems) and/or IL-10 (10 μg/ml, BD) were added.

### Quantitative RT-PCR

2 ×10^5^ Naïve B cells were cultured for 6 days on 50,000 irradiated CD40L-expressing fibroblasts in the presence of 50 ng/ml recombinant mouse IL-21, 0,5 ng/ml rhTGF-β, 100 ng/ml rhAPRIL and 100 ng/ml rhBAFF. mRNA was extracted using the RNeasy Micro Kit (Qiagen) and reverse transcribed using the High-Capacity cDNA Reverse Transcription Kit (Applied Biosystems). Quantitative PCR was performed on a StepOnePlus Real-Time PCR System (Applied Biosystems) using SYBR green (Bio-Rad). Expression of the target gene was normalized to the expression of the housekeeping gene GAPDH. The following primers were used (5′- 3′): to detect Iα1-Cα1: Iα1 forward CTCAGCACTGCGGGCCCTCCA and Cα1 reverse GTTCCCATCTGGCTGGGTGCTGCA; Iα-Cμ: Iα forward CAGCAGCCCTCTTGGCAGGCAGCCAG and Cμ reverse AGACGAGGGGGAAAAGGGTT; to detect *AICDA:* forward AGAGGCGTGACAGTGCTACA and reverse TGTAGCGGAGGAAGAGCAAT (all derived from Sigma-Aldrich).

### Total RNA Isolation

Sorted naïve human B cells were cultured for 6 days in the presence of APRIL, TGF-β, BAFF or the combination of TGF-β and APRIL. After the 6 days, the naïve (IgD^+^CD27^−^) B cells were sorted from the cells that isotype switched to IgA (CD19^+^IgA^+^IgD^−^). Total RNA was extracted from at least 1 ×10^5^ cells using a RNeasy Micro Kit (74004; Qiagen) according to the kit instructions.

### RNA-Seq Library Generation and Sequencing

RNA concentration and integrity was measured using the Qubit RNA BR Assay kit (Life Technologies) and an Agilent Technologies 2100 Bioanalyzer, correspondingly. cDNA libraries were constructed with the Illumina TruSeq™ RNA Sample Preparation Kit (Illumina, Inc., San Diego, CA, USA) using 400 ng of total RNA with RNA integrity number (RIN) ≥7. Briefly, the protocol consisted of polyA-RNA enrichment, RNA fragmentation, reverse transcription of fragmented RNA into cDNA, adapters ligation onto both ends of the cDNA fragments and amplification of cDNA fragments by PCR. Resulting cDNA libraries were paired-end sequenced on Illumina HiSeq 4000 by Macrogen (Seoul, Korea) to obtain around 40 million reads per sample.

### RNA-Seq Data Analysis

Artifacts such as low quality reads, adaptor sequence, contaminant DNA and PCR duplicates were removed using Trimmomatic v0.32 (31). Trimmed reads were mapped to reference human genome (UCSC hg19 assembly) with TopHat v2.0.13 (32) using the Bowtie2 v2.2.3 algorithm (33) and then aggregated with htseq-count v0.9.1 (34) to give total read counts for each of the 26,012 gene features.

### Differential Expression Analysis

Differential expression (DE) analysis was performed with DESeq2 (35). Minimal pre-filtering of the read counts data was applied first, which excluded gene with sum of 0 or 1 counts across all samples. Library sizes were normalized by the relative log-expression (RLE) method (36). Outlier genes were removed from the analysis using Cook's distance and low-expressed genes were excluded automatically by independent filtering procedure in DESEq2 workflow. Negative binomial generalized linear model for each gene was fitted and Wald test was applied for significance testing. Obtained *p*-values were adjusted by Benjamini-Hochberg method to control false discovery rate (FDR). Genes with adjusted *p*-values <0.01 were considered as DE genes. For heatmap visualization and clustering regularized log-transformation (rlog) of the read counts was applied and relative amount by which each gene expression deviates in a specific sample from gene's average across all samples was plotted. Data analysis of DE genes was conducted in R 3.3.3 (https://www.r-project.org) by Biobelka Genomics (the Netherlands).

### ELISA

Cytokine levels in supernatants were measured by ELISA, using antibody pairs for the following cytokines: TNF, TGF-β (both eBioscience) and IL-10 (BD Pharmingen).

### Statistics

All statistical analyses were performed using Prism (GraphPad Software). ^*^P < 0.05, ^**^P < 0.01, and ^***^P < 0.001 were considered statistical significant.

## Results

### IL-10-Producing Human B Cells Preferentially Belong to the IgA^+^ Subset

**Figure 1:**
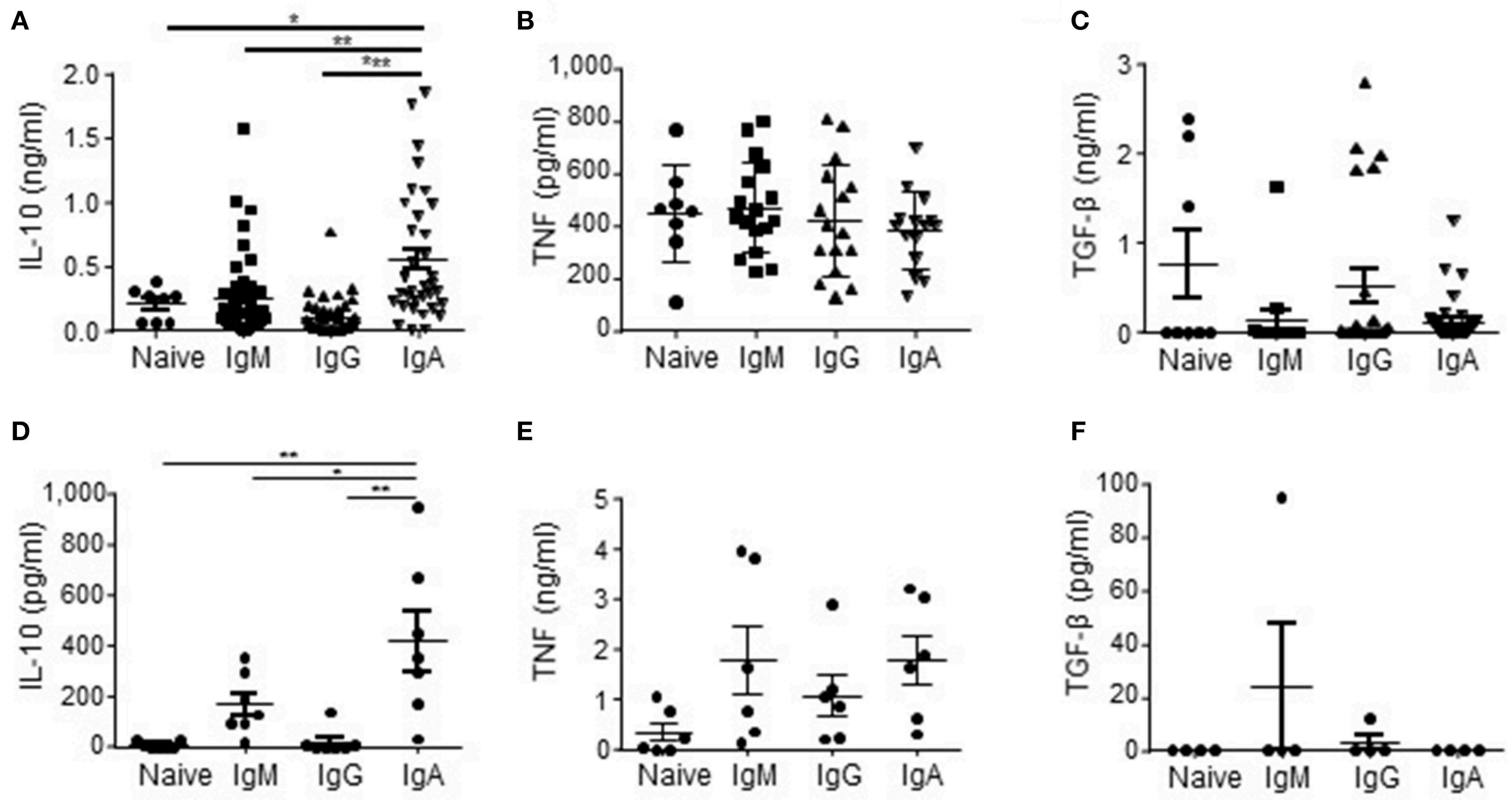
IL-10 is preferentially produced by human IgA^+^ B cells. **(A–C)** In order to rest the B cells, naïve (CD19^+^IgD^+^CD27^−^; *n* = 8), IgM (CD19^+^CD27^+^IgM^+^; *n* = 45), IgG (CD19^+^CD27^+^IgG^+^; *n* = 41), and IgA (CD19^+^CD27^+^IgA^+^; *n* = 35) human B cell clones were cultured for 48 h in IMDM supplemented with 10% FCS in the absence of CD40L-expressing fibroblasts and IL-21. In the final 5 h of the culture PMA/IO was added, where after supernatants were harvested and analyzed for the presence of IL-10 **(A)**, TNF **(B)**, and TGF-β **(C)** using ELISA. Each dot represents an individual B cell clone derived from peripheral blood of two healthy donors. **(D–F)** Freshly isolated peripheral blood-derived naïve (CD19^+^IgD^+^CD27^−^), IgM (CD19^+^CD27^+^IgM^+^), IgG (CD19^+^CD27^+^IgG^+^), and IgA (CD19^+^CD27^+^IgA^+^) human B cells were cultured for 16 h in IMDM containing 10% FCS. In the final 5 h of the culture PMA/IO was added, where after supernatants were harvested and analyzed for the presence of IL-10 **(D)**, TNF **(E)** and TGF-β **(F)** using ELISA. Data are shown for *n* = 7 healthy donors **(D)**, *n* = 6 donors **(E)** and *n* = 4 donors **(F)**. Mean ± SD are shown. ^*^P < 0.05, ^**^P < 0.01, and ^***^P < 0.001 (one-way ANOVA with Bonferroni multiple comparison test).

As APRIL is known to induce IgA class switching (37–39), we started to investigate our hypothesis that APRIL drives IL-10-producing Breg cells (27) by assessing if human IgA^+^ B cells had an increased potential to produce IL-10 compared to other B cell subsets. To this end we generated a library of B cell clones through immortalization of B cells derived from healthy donor peripheral blood using transduction of Bcl6 and Bcl-xL. We observed that in this B cell library the percentage of IgA^+^ B cell clones producing high levels of IL-10 upon *in vitro* stimulation with PMA/ionomycine (IO) was increased compared to B cell clones with a naïve (defined as CD19^+^IgM^+^CD27^−^), memory IgM (CD19^+^IgM^+^CD27^+^), or memory IgG (CD19^+^IgG^+^CD27^+^) phenotype before transduction: 60% of the IgA^+^ B cell clones produced more than 288 pg/ml of IL-10, which is the average amount of IL-10 produced by all the clones, compared to 25% (*p* = 0.11), 29% (*p* = 0.007) and 8% (*p* = 1 ×10^−6^) of the naïve, IgM^+^ and IgG^+^ B cell clones respectively (Figure 1A) The IgA^+^ B cell clones produced on average 556 pg/ml IL-10 vs. 208 pg/ml (*p* = 0.04), 257 pg/ml (*p* = 0.0015) and 104 pg/ml (*p* = 4.4 ×10^−7^) of IL-10 produced by the naïve, IgM and IgG B cell clones, respectively (Figure 1A). Of note, the IgA^+^ B cell clones produced even without PMA/IO stimulation significantly higher levels of IL-10 compared to the other types of B cells clones: on average the IgA^+^ B cell clones produced 142 pg/ml of IL-10 vs. 24 pg/ml (*p* = 0.026), 4 pg/ml (*p* = 0.003), and 2 pg/ml (*p* = 0.003) of IL-10 produced by the naïve, IgM and IgG B cell clones, respectively (data not shown). In contrast, no differences in the frequencies of TNF and TGF-β producing B cells were observed between the different B cell subsets (Figures 1B,C).

The initial observation using B cell clones was confirmed using freshly isolated primary FACS-sorted bulk naïve, memory IgM^+^, IgG^+^, and IgA^+^ B cells derived from peripheral blood of healthy donors. These primary IgA^+^ B cells produced on average 424 pg/ml IL-10, whereas we measured 7, 170, and 17 pg/ml for the naïve, memory IgM^+^ and IgG^+^ B cells, respectively (Figure 1D). No significant different amounts of TNF and TGF-β were produced by the primary B cell subsets (Figures 1E,F), indicating that human IgA^+^ B cells are specifically prone to secrete IL-10.

### APRIL, but Not TGF-β and BAFF, Promotes the Differentiation of Naïve B Cells to IL-10-Producing IgA^+^ B Cells

**Figure 2:**
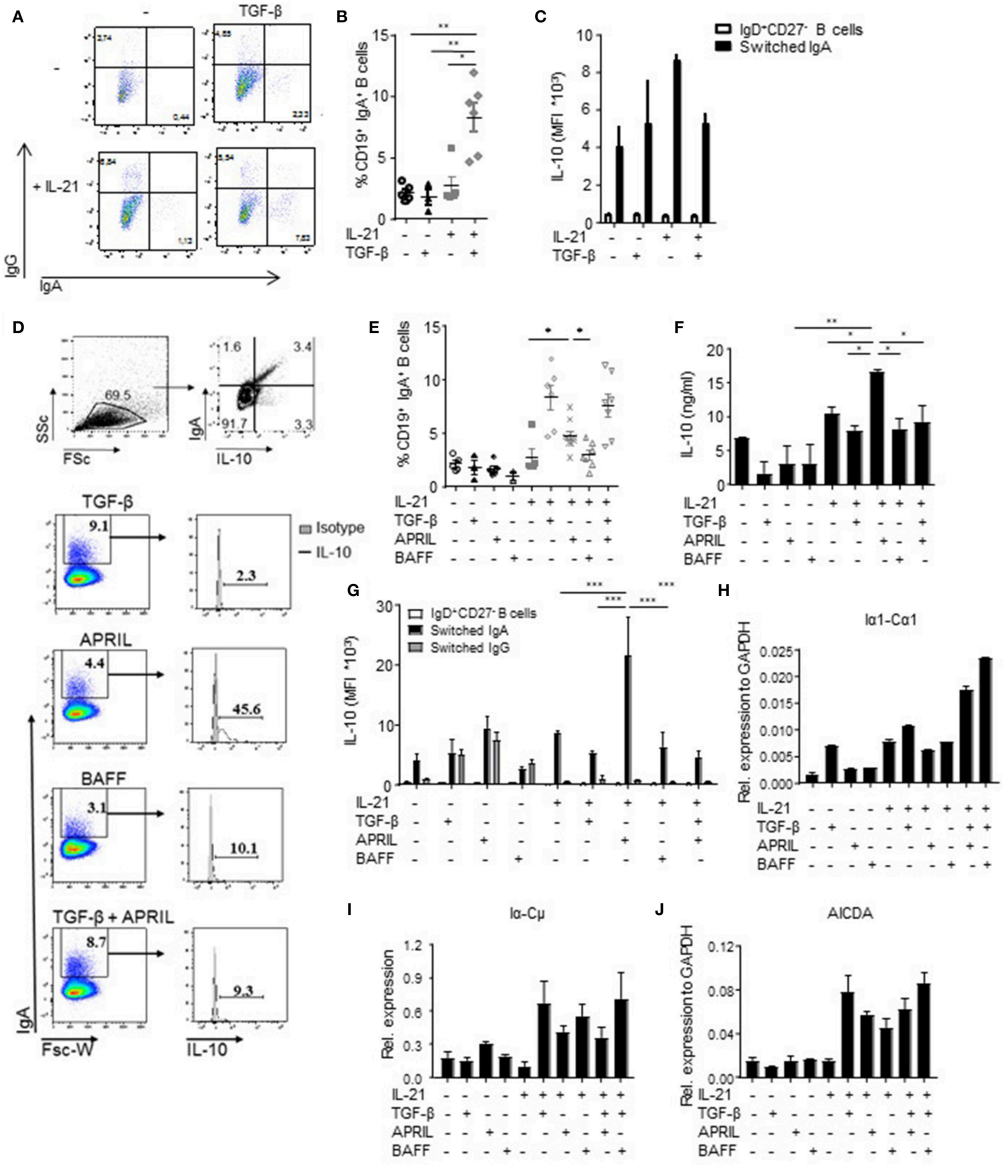
APRIL is an important factor to induce CSR to IgA. **(A)** Representative dot plots of class-switched IgG^+^ and IgA^+^ CD19^+^ B cells at day 6 of culture in the presence of CD40L-expressing fibroblasts and IL-21 or TGF-β, or a combination of these factors. **(B)** Frequencies of CD19^+^IgA^+^ B cells after 6 days. *N* = 4-6, mean ± SD are shown. ^*^P < 0.05, ^**^P < 0.01 (one-way ANOVA with Bonferroni multiple comparison test). **(C)** IL-10 production by naïve and class switched B cells cultured in the presence of IL-21, TGF-β or an combination, as measured by flow cytometric analyses of double-stained cells for IgA and IL-10. Mean ± SD are shown, *n* = 5. **(D)** Representative dot plots (upper part) and histograms (lower part) of class-switched IL-10-producing IgA^+^ B cells at day 6 of culture in the presence of CD40L-expressing fibroblasts, IL-21 and indicated stimuli. Frequencies of IgA^+^ B cells and IL-10-producing B cells within the IgA^+^ subsets are shown. (E) Frequencies of CD19^+^IgA^+^ B cells after 6 days. *N* = 4-7, mean ± SD are shown. ^*^P < 0.05 (one-way ANOVA with Bonferroni multiple comparison test). **(F)** Detection of IL-10 by ELISA in the supernatants of stimulated B cells at day 6 of the culture. Mean ± SD are shown, *n* = 5. ^*^P < 0.05, ^**^P < 0.01 (one-way ANOVA with Bonferroni multiple comparison test). **(G)** APRIL-induced IgA^+^ B cells produced high amounts of IL-10 after 6 days of culture without additional stimulation like CpG or PMA/IO, as measured by flow cytometric analyses of double-stained cells for IgA and IL-10. Mean ± SD are shown, *n* = 5. ^***^P < 0.001 (two-way ANOVA). **(H–J)** Recent class switching to IgA was detected by the presence of mRNA transcripts for Iα1-Cα1 **(H)**, Iα-Cμ **(I)**, and *AICDA* **(J)** measured by quantitative real time RT-PCR. Mean ± SD are shown, *n* = 3.

Since we observed that not all human IgA^+^ B cells produced IL-10 (Figures 1A,D), we hypothesized that IL-10 production was a unique property of a distinct subset of IgA^+^ B cells. Therefore, we investigated which factors driving IgA isotype switching also induced IL-10 production. In line with literature, the combined administration of TGF-β and IL-21 to FACS-sorted human naïve B cells cultured on CD40L-expressing fibroblasts induced CSR toward IgA (40, 41) (Figures 2A,B); however, this cytokine cocktail failed to induce IL-10 production (Figure 2C), suggesting that although TGF-β promoted the differentiation of naïve B cells toward IgA^+^ B cells, it fails to induce IL-10 production. BAFF + IL-21 had a modest effect on IgA class switching compared to IL-21 alone (Figure 2E) and also did not induce significant IL-10 secretion (Figures 2D,F,G). In contrast, APRIL + IL-21 not only promoted isotype switching to IgA (Figures 2D,E), but also induced IL-10 secretion (Figures 2D,F), with cells cultured in the presence of APRIL and IL-21 releasing significantly more IL-10 than cells cultured in the presence of IL-21 alone, IL-21+TGF-β, or IL-21+BAFF (Figures 2D,F). Of note, stimulation of B cells with IL-21+APRIL+TGF-β effectively induced CSR to IgA (Figure 2E), but decreased IL-10 production by the IgA^+^ B cells compared to IL-21+APRIL alone (Figures 2D,F). Of note, more than 75% of the IL-21+APRIL-induced IgA^+^ B cells are IgA1, whereas a small proportion belong to the IgA2 isotype (Supplementary Figure 1). Neither TGF-β, APRIL, or BAFF induced IgA isotype switching or IL-10 secretion in the absence of IL-21 (Figures 2E,F), most probably because IL-21 is required to drive proliferation and thereby facilitates isotype switching (Supplementary Figure 2) (42, 43). Therefore, IL-21 was added to all experimental conditions in the subsequent experiments.

To confirm the observations that the IL-10 was specifically produced by the B cells that have class switched to IgA, we performed an IL-10 catch assay followed by flow cytometry. IL-10 production was specifically and significantly increased in APRIL-induced IgA^+^ B cells but not in IgD^+^CD27^−^ B cells or IgG^+^ B cells present in the same culture (Figure 2G). Also in the TGF-β, BAFF, and TGF-β+APRIL culture conditions, IL-10 production was restricted to IgA^+^ B cells, but the IL-10 signal was not increased in comparison with the IL-21 only culture condition and significantly lower than in the APRIL-stimulated IgA^+^ B cells (Figure 2G).

The IgA^+^ B cells present in our experiments could have originated from active and recent CSR to IgA of naïve B cells rather than from expansion of pre-existing IgA^+^ cells potentially contaminating the naïve B cells at the start of the experiment. In experiments addressing this question we found that upregulation of surface IgA was associated with the presence of Iα-Cμ switch circle transcripts, upregulated Iα1-Cα1 germline transcription, and upregulated transcription of *AICDA*, the gene encoding for AID (Figures 2H–J). In line with these data, direct APRIL stimulation of peripheral blood-derived IgA^+^ B cells that had already class-switched to IgA *in vivo* failed to increase the amounts of IL-10 secreted by these cells (Supplementary Figure 3). Hence, we conclude that TGF-β, APRIL, and BAFF are all able to drive human naïve B cells to isotype switch to IgA in the presence of CD40L and IL-21, but that only APRIL + IL-21 induced a distinct population of IgA^+^ B cells producing high levels of IL-10.

### APRIL-Induced CSR to IgA Is Not Affected by Autocrine IL-10 and TGF-β Signaling

**Figure 3:**
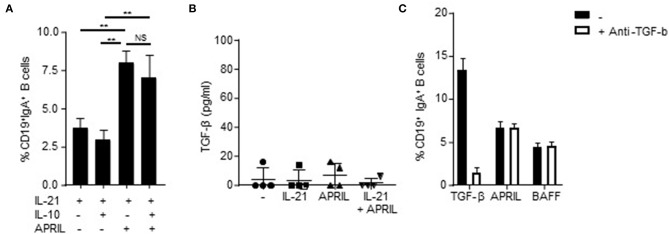
APRIL-induced CSR to IgA is independent of autocrine TGF-β or IL-10 signaling. **(A)** Percentage of class-switched IgA^+^ B cells at day 6 of culture in the presence of CD40L-expressing fibroblasts, IL-21, and indicated stimuli. Mean ± SD are shown, *n* = 3. ^**^P < 0.01 and NS: not significant (one- or two-way ANOVA with Bonferroni multiple comparison test). **(B)** Human naïve B cells were cultured for 3 days in the presence of CD40L-expressing fibroblasts and indicated stimuli, whereafter the culture supernatant was harvested and TGF-β was detected using ELISA. *N* = 4. **(C)** Naïve human B cells were cultured for 6 days in the presence of CD40L-expressing fibroblast, IL-21 and indicated stimuli. Neutralizing antibodies to TGF-β (100 ng/ml) were added at day 0 and 3 of the culture. After 6 days, the B cells were harvested and the percentage of class-switched IgA^+^ B cells was detected using flow cytometry. Mean ± SD are shown, *n* = 3.

Since IL-10 itself has been reported as a factor involved in CSR to IgA (44), we analyzed whether addition of IL-10 to the APRIL-induced IgA^+^ B cells is important for the development and/or maintenance of these cells. APRIL-induced CSR to IgA is not dependent on signaling via IL-10, as we could not detect an additive effect of IL-10 on the percentages of class-switched cells after stimulation with APRIL (Figure 3A). Moreover, we also assessed whether APRIL induces CSR to IgA through a mechanism involving autocrine production of TGF-β. No autocrine TGF-β could be detected in the supernatant of CD40L-stimulated naïve B cells stimulated with IL-21, APRIL or IL-21+APRIL (Figure 3B). Furthermore, addition of neutralizing antibodies did not affect the percentages of class switched IgA^+^ B cells stimulated by APRIL or BAFF (Figure 3C). As expected, neutralization of TGF-β significantly reduced CSR to IgA in the cultures stimulated with TGF-β. Collectively, these results indicate that APRIL-induced CSR to IgA is not dependent on autocrine IL-10 or TGF-β signaling.

### APRIL-Induced IgA^+^ B Cells Display a Regulatory B Cell Phenotype

**Figure 4:**
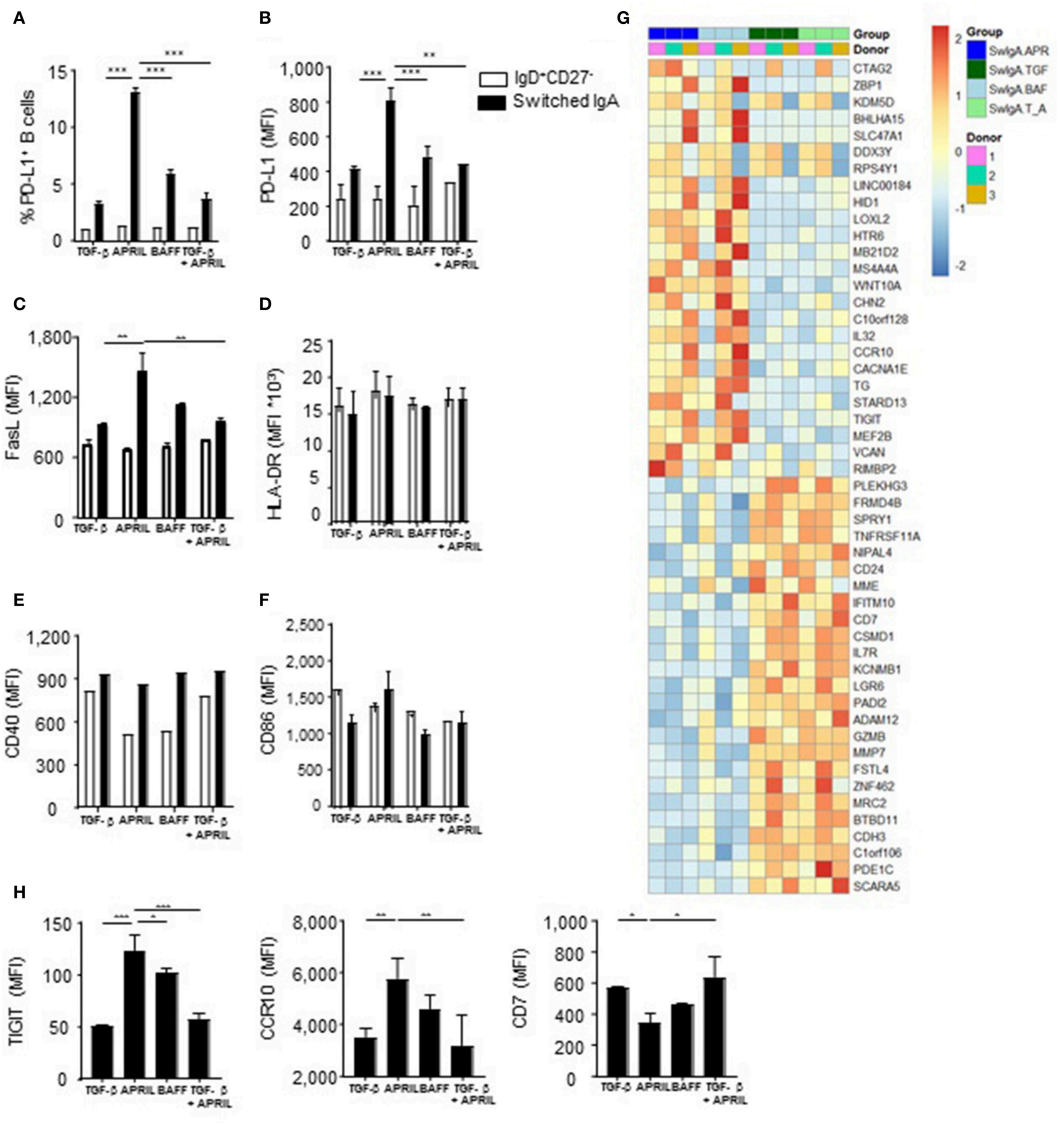
APRIL-induced IgA^+^ human B cells obtain a regulatory B cell phenotype by expressing high levels of IL-10, PD-L1, FasL and TIGIT. **(A–H)** A panel of co-stimulatory and inhibitory molecules as well as HLA-DR was assessed on IgD^+^CD27^−^ and class switched IgA^+^ B cells after 6 days of culture on CD40L-expressing fibroblasts and IL-21 supplemented with TGF-β, APRIL, BAFF or a TGF-β+APRIL. **(A)** The frequency of PD-L1^+^CD19^+^ B cells is shown. MFI of PD-L1 **(B)**, FasL **(C)**, HLA-DR **(D)**, CD40 **(E)**, CD86 **(F)**, and TIGIT, CCR10, and CD7 **(H)** was analyzed on naïve and class switched IgA^+^ B cells. **(G)** Heatmap of relative expression of the top 25 most up- and downregulated genes in APRIL, TGF-β, BAFF or TGF-β+APRIL (SwIgA T_A) stimulated class switched IgA^+^ B cells. Naïve B cells were cultured for 6 days on CD40L-expressing fibroblasts and IL-21 supplemented with TGF-β, APRIL, BAFF or a TGF-β+APRIL where after class switched IgA^+^ were sorted and RNA was isolated. *N* = 3 donors per stimulation. **(A–H)** Mean ± SD are shown, *n* = 3. ^*^P < 0.05, ^**^P < 0.01 and ^***^P < 0.001 (two-way ANOVA with Bonferroni multiple comparison test).

Since IL-10 production is a hallmark cytokine produced by regulatory B cells (4), we assessed the expression of a number of other key co-stimulatory and immune-inhibitory molecules associated with regulatory B cells, including PD-L1 and Fas ligand (FasL) (45, 46). There was a significant 4-fold increase in PD-L1 positive cells in the APRIL-induced IgA^+^ cells compared to the TGF-β- or BAFF-induced IgA^+^ B cells, which was paralleled by higher number of PD-L1 molecules per cell as assessed by MFI (Figures 4A,B). Similarly, FasL was significantly upregulated in the APRIL-induced IgA^+^ cells as compared to the TGF-β- or BAFF-induced IgA^+^ B cells (Figure 4C). Strikingly, the addition of TGF-β to APRIL completely abrogated the increased expression of PD-L1 and FasL on the class switched IgA^+^ B cells (Figures 4A–C), similar to the impact on IL-10 (Figures 2F,G) and indicating that TGF-β interferes with the induction of the IgA^+^ B cell regulatory phenotype by APRIL. No differences were found in the expression levels of the co-stimulatory molecules CD40 and CD86, and of HLA-DR (Figures 4D–F).

In order to confirm that APRIL induces a distinct IgA^+^ B cell phenotype on a broader molecular level, we performed a whole transcriptome analysis of IgA^+^ B cells generated with APRIL, TGF-β, TGF-β+APRIL, or BAFF. APRIL-induced IgA^+^ B cells were clearly distinct from TGF-β-induced IgA^+^ B cells, with a total of 447 differentially regulated transcripts (Supplementary Table 1 lists 103 upregulated genes and 63 downregulated genes with a fold change > 1 or < −1, respectively). Similarly, we identified a total of 301 differentially regulated transcripts (154 upregulated and 147 downregulated) between APRIL-induced IgA^+^ B cells and the TGF-β+APRIL-induced IgA^+^ B cells (Supplementary Table 2 lists 80 upregulated genes and 58 downregulated genes with a fold change > 1 or < −1, respectively). Accordingly, a cluster analysis on the top 25 up- and downregulated genes showed that the APRIL-induced IgA^+^ B cells were clearly distinct from the TGF-β and TGF-β+APRIL induced IgA^+^ B cells, with the latter two showing similar profiles (Figure 4G). We confirmed these transcriptional data by FACS analysis of protein expression of 3 target genes: expression of TIGIT, CCR10, and CD7 was significantly altered on APRIL-induced IgA^+^ B cells in comparison with TGF-β-induced and TGF-β+APRIL-induced IgA^+^ B cells (Figure 4H).

Intriguingly, the transcriptional analysis did not reveal any differentially expressed gene between APRIL-induced and BAFF-induced IgA^+^ B cells (Figure 4G). At the protein level, however, a significant higher protein expression of TIGIT [a molecule which has been associated with immunoregulation (47)] was observed on APRIL-induced IgA^+^ B cells compared to BAFF-induced IgA^+^ B cells. Collectively, these data indicate that APRIL-induced IgA^+^ B cells have a unique regulatory phenotype extending beyond IL-10, which is not observed on other IgA^+^ B cell subsets.

### APRIL-Induced IgA^+^ IL-10^+^ B Cells Are Phenotypically Distinct From Other Breg Subsets

Since IL-10 production is the hallmark of previously described human Breg subsets such as CD19^+^CD24^hi^CD38^hi^CD1d^+^ transitional B cells, CD24^hi^CD27^+^ memory B cells, and CD24^−^CD27^hi^CD38^hi^ plasmablasts, we analyzed to what extend the APRIL-driven IgA^+^ Bregs expressed similar phenotypic markers. Interestingly, the majority of the APRIL-driven IgA^+^ Bregs did not upregulate expression of CD24, CD27, CD38, CD1d, CD5 or CD138 after 6 days of culture in the presence of IL-21 and APRIL (Supplementary Figures 4A–C). No differences were observed between the B cells that retained a naïve phenotype (CD19^+^CD27^−^IgD^+^) during the 6 days culture period in the presence of IL-21 and APRIL and the APRIL-induced class switched IgA^+^ Bregs with regard to the expression of markers on CD24^hi^CD27^+^ memory B cells or on CD24^−^CD27^hi^CD38^hi^ plasmablasts (Supplementary Figures 4A,B). Therefore, the APRIL-induced IgA^+^ IL10^+^ B cells appear to be distinct from previously described IL-10 producing human Bregs.

Recently it was shown that a large fraction of IgM^+^ memory and IgA^+^ B cells express the gut-homing markers α4β7 and CCR9 (48). These authors also show that IL-10, critical for gut homeostasis, is enriched in intestinal IgM^+^ and IgA^+^ B cells. Here we assessed whether the IL-10-producing IgM^+^ and IgA^+^ B cells directly isolated from blood and the APRIL-induced class switched IgA^+^ B cells express α4β7 and CCR9. Human peripheral blood-derived IgA^+^ B cells do express α4β7, however, expression of α4β7 is the highest on APRIL-induced IgA^+^ Bregs (Supplementary Figure 5). None of the cells expressed CCR9. These data suggest that APRIL-induced IgA^+^ Bregs can acquire the potential to home to the gut.

### The APRIL-Induced Regulatory B Cell Phenotype Requires TACI Signaling and Is Stable Upon B Cell Activation

**Figure 5:**
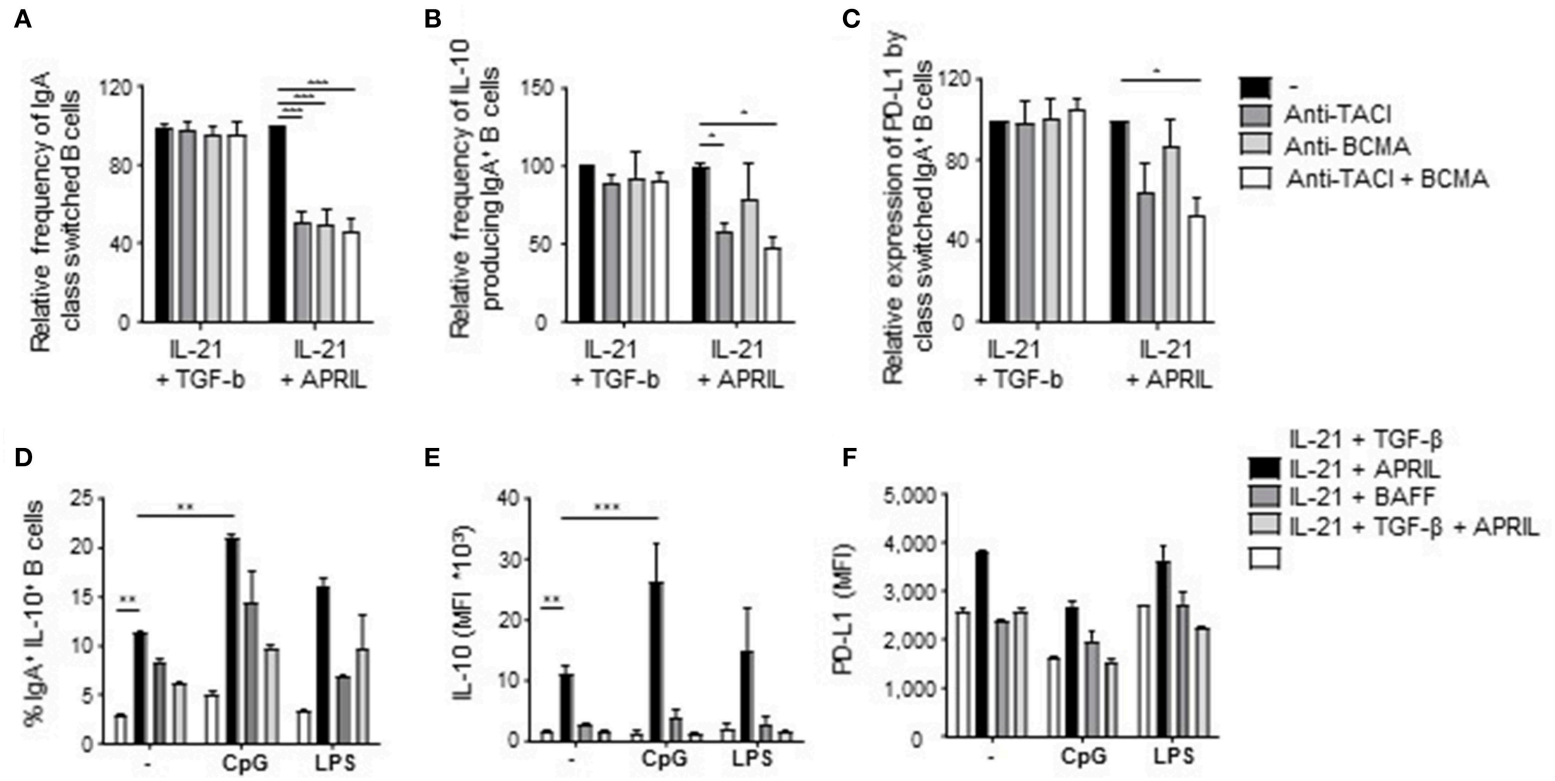
Signaling via TACI is required for the induction of APRIL-driven regulatory IgA^+^ B cells. Peripheral blood-derived naïve (CD19^+^IgD^+^CD27^−^) B cells were cultured for 6 days in the presence of CD40L-expressing fibroblasts and IL-21 + TGF-β or APRIL. Blocking antibodies to TACI and/or BCMA were added to the cultures. After 6 days, class switching to IgA was analyzed using flow cytometry. **(A)** Relative frequencies of class switched IgA^+^ in the presence of blocking antibodies to TACI and/or BCMA are shown. Mean ± SD are shown, *n* = 3. **(B,C)** The regulatory phenotype of the class switched IgA^+^ B cells cultured in the presence of blocking antibodies to TACI and/or BCMA was assessed after 6 days of culture by analyzing the frequency of IL-10^+^IgA^+^ B cells **(B)** and the MFI of PD-L1 **(C)** using flow cytometry. **(A–C)** Data are normalized to the controls for each dataset (IL-21+TGF-β s. IL-21+APRIL) separately. Mean ± SD are shown, *n* = 3. ^*^P < 0.05 and ^***^P < 0.001 (one-way ANOVA with Bonferroni multiple comparison test). **(D–F)** The intrinsic regulatory phenotype of APRIL-induced IgA^+^ B cells is enhanced in the presence of CpG. The frequency of class switched IgA^+^ B cells (D) and the expression of IL-10 **(E)** and PD-L1 **(F)** were assessed using flow cytometry. Mean ± SD are shown, *n* = 3. ^**^P < 0.01 and ^***^P < 0.001 (two-way ANOVA with Bonferroni multiple comparison test).

To explore further which factors influence this unique APRIL-induced Breg phenotype, we first analyzed via which receptor APRIL triggered its effects. APRIL can bind to BCMA and to TACI, which are expressed on B cells and activated T cells, respectively(17, 18, 49–51). Signaling via TACI and BCMA was important for the induction and/or survival of APRIL-induced IgA^+^ Bregs, since addition of TACI- and BCMA-specific blocking antibodies diminished the frequency of class switched IgA^+^ B cells (Figure 5A). In contrast, only the blockade of TACI resulted in a decrease in IL-10 and PD-L1 on APRIL-mediated class switched IgA^+^ B cells (Figures 5B,C). As anticipated, no effects of the blocking antibodies were observed when B cells were stimulated with TGF-β (Figures 5A–C). Recently is has been described that TACI signaling activates the mechanistic target of rapamycin (mTOR) pathway, leading to immunoglobulin class switching and plasmablast differentiation (52). Here, we investigated whether APRIL induces CSR to IgA via this TACI-mediated mTOR pathway. As shown in Supplementary Figure 6, activation of the mTOR pathway is required for IgA CSR, since addition of rapamycin inhibits CSR to IgA. However, activation of the mTOR pathway is not essential for IL-10 production by the class switched IgA^+^ B cells under these conditions.

We next assessed whether the regulatory phenotype of APRIL-induced IgA^+^ B cells was maintained and/or enhanced upon activation as, in contrast to previously described Breg subsets, these cells produced significant IL-10 amounts in the absence of additional activation in our culture conditions. In line with literature, CpG stimulation enhanced the frequency of IgA^+^IL-10^+^ class switched B cells upon APRIL stimulation (Figure 5D). Also, the amounts of IL-10 produced per APRIL-induced IgA^+^ regulatory B cell were significantly increased upon stimulation with CpG (Figure 5E), whereas the expression levels of PD-L1 did not change (Figure 5F). In addition, the expression of IL-10 or PD-L1 by APRIL-induced IgA^+^ regulatory B cells remained stable upon LPS stimulation, indicating that APRIL-induced regulatory B cell phenotype is stable upon activation. Collectively, these data indicate that the induction of the Breg phenotype of APRIL-induced IgA^+^ B cells is dependent on TACI signaling but does not require additional B cell activating signals.

### APRIL-Induced IgA^+^ Regulatory B Cells Inhibit T Cell Responses *in vitro*

**Figure 6:**
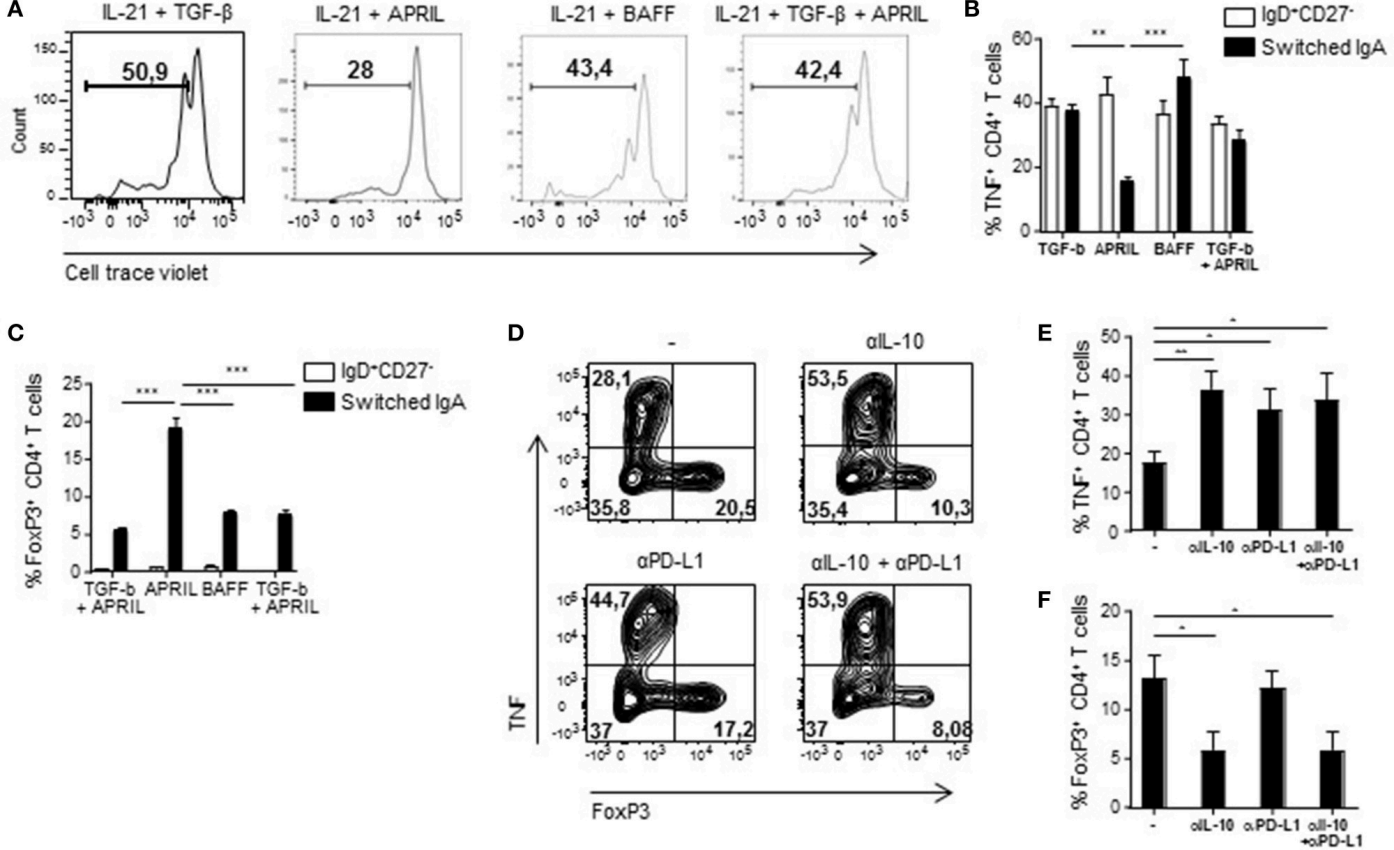
APRIL-induced IgA^+^ regulatory B cells suppress CD4^+^ T cell proliferation and TNF production, while stimulating the expansion of FoxP3^+^CD4^+^ T cells. **(A)** After 5 days of co-culture of Bregs and autologous CD4^+^ T cells, proliferation of the CD4^+^ T cells was analyzed using a cell trace violet dye and flow cytometry. Representative histograms are shown. **(B)** TNF production by the CD4^+^ T cells was assessed after 3 days of co-culture using flow cytometry. Percentages of TNF^+^CD4^+^ T cells are depicted. **(C)** The frequency of regulatory FoxP3^+^CD4^+^ T cells was analyzed after 3 days of co-culture using flow cytometry. **(D–F)** Neutralizing antibodies to IL-10 and PD-L1 were added to the co-cultures. After 3 days of co-culture of APRIL-induced IgA^+^ Bregs and CD4^+^ T cells in the presence of neutralizing antibodies to IL-10, PD-L1, the combination or control medium, the frequencies of TNF^+^ and FoxP3^+^ CD4^+^ T cells were assessed. **(D)** Representative dot plots of the frequencies of TNF^+^ and FoxP3^+^ CD4^+^ T cells. **(E,F)** Quantification of the frequencies of TNF^+^ **(E)** and FoxP3^+^ **(F)** CD4^+^ T cells induced by APRIL-driven regulatory IgA^+^ B cells. **(B–F)** Mean ± SD are shown, *n* = 4. ^*^P < 0.05, ^**^P < 0.01, and ^***^P < 0.001 (one-way ANOVA with Bonferroni multiple comparison test).

In order to assess if the regulatory phenotype of APRIL-induced IgA^+^ B cells translates into functional suppressive capacities, we measured proliferation and T cell polarization following co-cultured of these B cells with autologous CD4^+^ T cells. CD4^+^ T cell proliferation was specifically reduced in the presence of APRIL-induced IgA^+^ regulatory B cells compared to TGF-β-, BAFF-, or TGF-β+APRIL-induced IgA^+^ B cells (Figure 6A). Whereas, no differences were observed in T cell skewing toward Th1, Th2, or Th17 T cells (data not shown), a significant decrease in TNF production by the T cells and a 4-fold increase in FoxP3^+^ T cells were observed in the presence of APRIL-induced IgA^+^ regulatory B cells (Figures 6B,C). To investigate the mechanism of suppression of TNF production and induction of FoxP3^+^ regulatory T cells, we added neutralizing antibodies to IL-10 and PD-L1 to the APRIL-induced IgA^+^ regulatory B cell: CD4^+^ T cell co-cultures. Inhibition of TNF production by the CD4^+^ T cells was mediated via IL-10 and PD-L1 (Figures 6D,E), whereas the induction of the FoxP3^+^ regulatory T cells was solely mediated via IL-10 (Figures 6D,F). Thus, we conclude that APRIL-induced IgA^+^ regulatory B cells inhibit CD4^+^ T cell proliferation and induce FoxP3^+^ regulatory T cells via IL-10 and PD-L1.

### APRIL-Induced IgA^+^ Regulatory B Cells Inhibit Macrophage Responses *in vitro*

**Figure 7:**
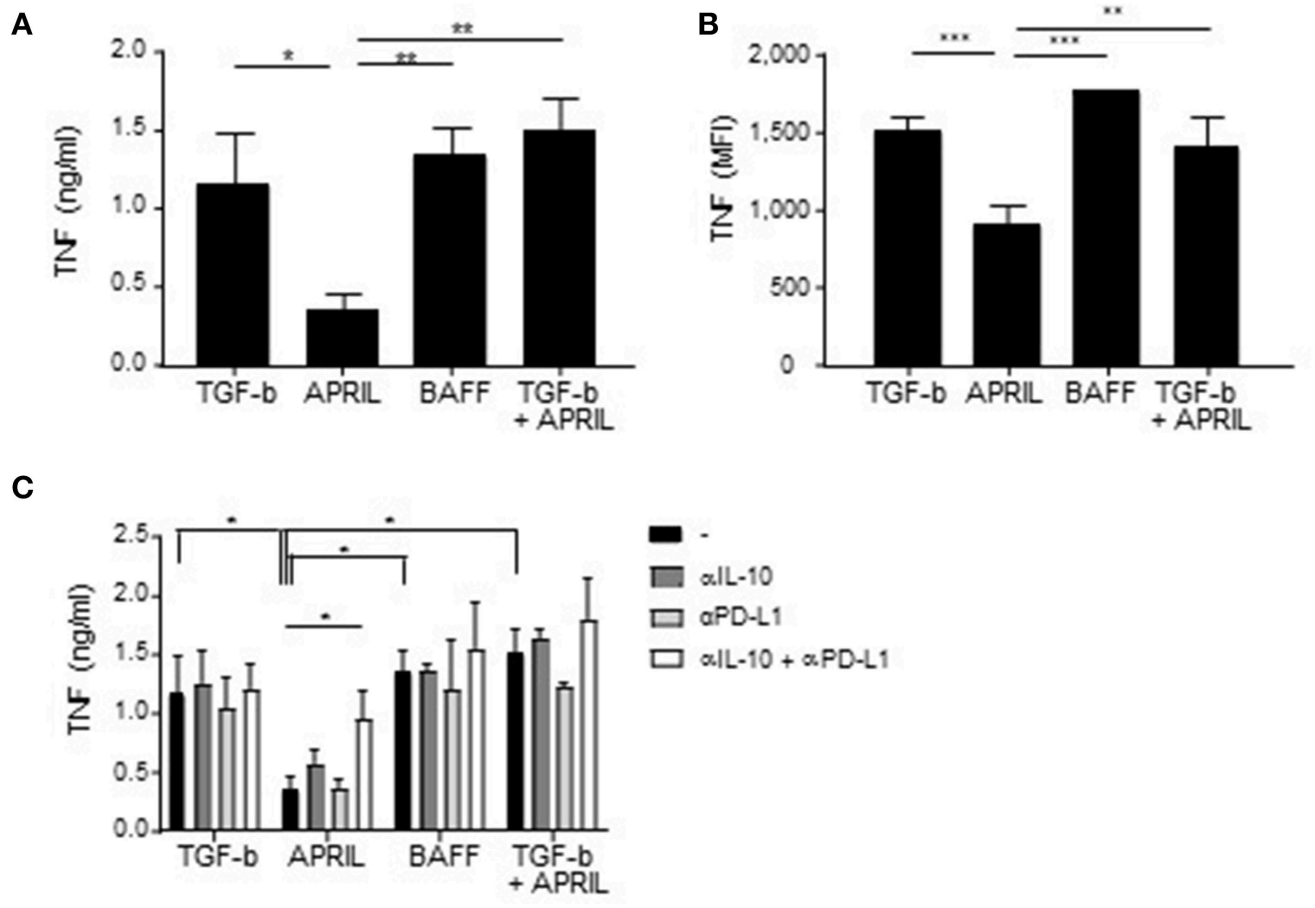
APRIL-induced IgA^+^ regulatory B cells suppress TNF production by GM-CSF macrophages. **(A,B)** TNF production by the macrophages upon co-culture with TGF-β-, APRIL-, BAFF- or TGF-β+APRIL-induced IgA^+^ B cells is assessed using TNF ELISA **(A)** and intracellular TNF staining **(B)**. **(C)** Neutralizing antibodies to IL-10 and PD-L1 were added to the co-cultures. After 3 days of co-culture, supernatant was harvested and TNF was measured using ELISA. Mean ± SD are shown, *n* = 3. ^*^P < 0.05, ^**^P < 0.01, and ^***^P < 0.001 (one- or two-way ANOVA with Bonferroni multiple comparison test).

To confirm and extend the functional relevance of the APRIL-induced IgA^+^ Bregs, we next assessed their potential to dampen the activation of myeloid cells. To this end, autologous pro-inflammatory GM-CSF macrophages or alternatively-activated M-CSF macrophages were co-cultured with FACS-sorted class switched IgA^+^ B cells in a 4:1 ratio. Significant lower amounts of TNF were detected when LPS-stimulated GM-CSF macrophages and M-CSF macrophages were cultured in the presence of APRIL-induced IgA^+^ regulatory B cells compared to TGF-β-, BAFF- or TGF-β+APRIL-induced IgA^+^ B cells (Figure 7A and data not shown, respectively). Since B cells can also produce TNF and could contribute to the effects measured in the supernatants, we analyzed TNF production by the macrophages specifically using flow cytometry. Again, significant lower amounts of TNF were detected when GM-CSF macrophages and M-CSF macrophages were cultured in the presence of APRIL-induced IgA^+^ regulatory B cells (Figure 7B). Both IL-10 and PD-L1 expression by the APRIL-induced IgA^+^ regulatory B cells were required to dampen TNF production by the GM-CSF macrophages (Figure 7C).

### APRIL-Driven Amelioration of EAE and CHS Is Associated With the Induction of IL-10-Producing Regulatory B Cells

**Figure 8:**
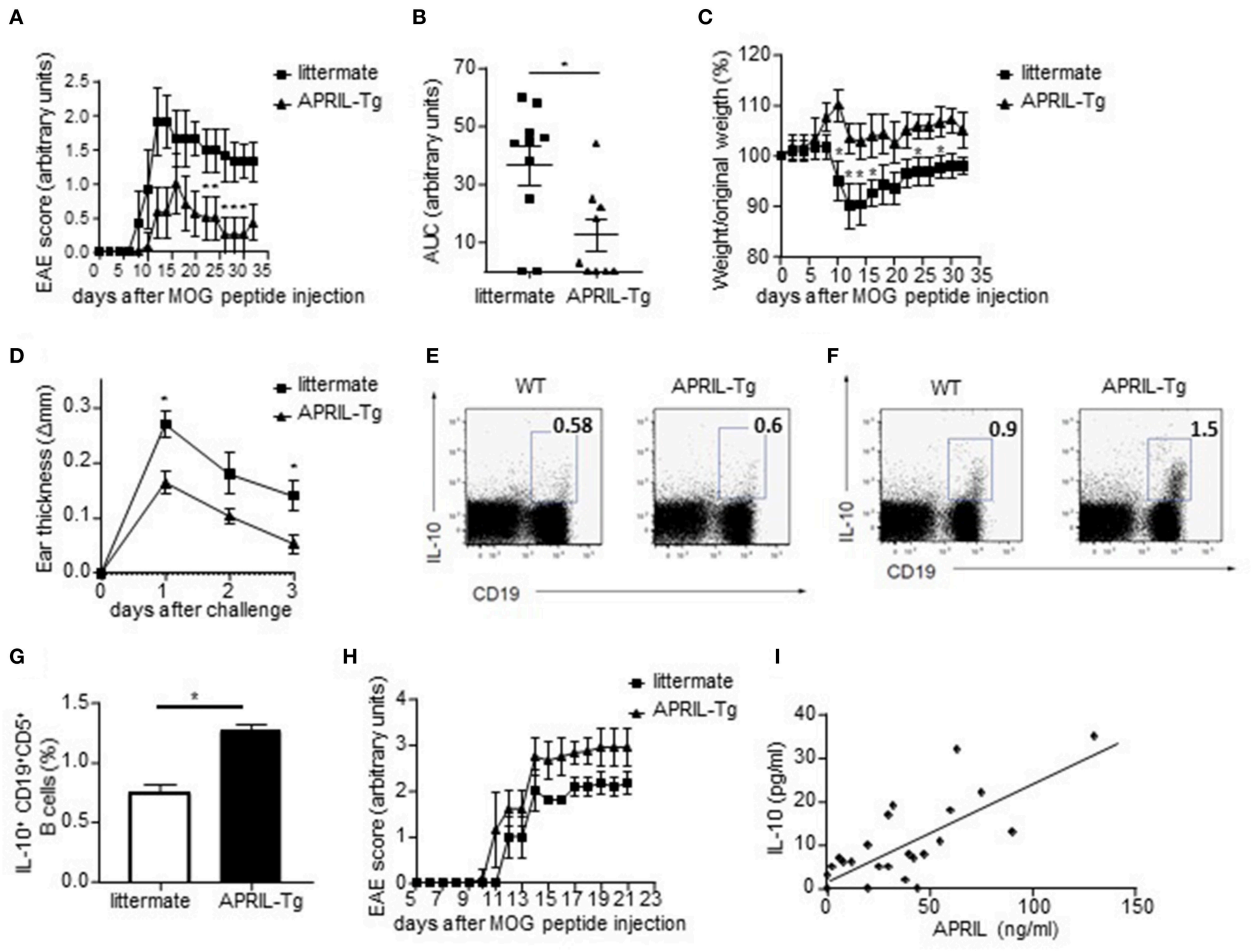
APRIL driven amelioration of EAE and CHS is associated with the induction of IL-10-producing regulatory B cells. **(A–C)** EAE was induced in APRIL transgenic (*n* = 10) and wild type mice (*n* = 9). APRIL-Tg mice had a decreased disease development, determined by disease score **(A)** and area under the curve for disease score **(B)**, and a lower weight loss compared to control mice **(C)**. The weight loss is given by the ratio of weight at the indicated time point vs. original weight. **(D–F)** Control and APRIL-Tg mice were immunized with oxazolone according to the CHS protocol. **(D)** Sensitized littermates (*n* = 4) and APRIL-Tg mice (*n* = 5) were challenged with oxazolone and ear thickness was measured 1, 2 and 3 days after the challenge. **(E)** No altered IL-10 production in splenocytes of unchallenged APRIL-Tg and control mice. A representative FACS staining is shown. **(F)** Representative FACS staining of IL-10 producing splenic B cells in APRIL-Tg and littermate mice 3 days after the challenge. **(G)** Quantitative analysis of peritoneal B cells for IL-10 production in sensitized mice 3 days after oxazolone challenge (*n* = 5). **(H)** Depletion of peritoneal B1 cells by hypotonic shock abolishes the disease modulating effect of ectopic APRIL expression in EAE. Distilled water-treated APRIL-Tg mice (*n* = 8) are equally susceptible to EAE as WT controls (*n* = 8). **(I)** Concentrations of APRIL and IL-10 were measured in the synovial fluids of patients with inflammatory arthritis (*n* = 24). Correlations were calculated using Spearman's rank order correlation. **(A–H)** Mean ± SD are shown, ^*^P < 0.05 (Mann-Whitney *U*-test).

We next assessed if the *in vitro* regulatory functions of APRIL-induced Bregs also translated *in vivo* using APRIL-tg mice. We have previously shown that these mice display a reduced incidence and severity of CIA compared to littermate controls, which was partially mediated through a decrease of collagen-specific autoantibodies (26). In order to assess whether APRIL exerts its anti-inflammatory effect not only through this mechanisms but also through the generation of IL-10-producing Bregs, we analyzed the effects of APRIL overexpression in two inflammatory disease models which are not critically dependent on autoantibodies. In MOG-induced EAE, where autoreactive B cells contribute to disease by activation of autoreactive T cells rather than by autoantibody production (1), the disease onset was similar in APRIL-Tg and littermate control mice, but APRIL-Tg mice manifested a significantly lower disease score and weight loss (Figures 8A–C). To confirm the results in a different antibody-independent inflammatory disease model, we assessed the effects of APRIL expression in a CHS model in which the animals were epicutaneously sensitized on the abdomen and challenged 5 days later at one ear with oxazolone. Ear swelling was maximal 24 h after challenge and gradually decreased afterwards (Figure 8D). Importantly, ear swelling was significantly lower in APRIL-Tg mice compared to controls (Figure 8D), indicating that APRIL strongly reduces inflammation in this model as well.

As these data suggest that APRIL can control inflammatory disease by other mechanisms than modulation of autoantibody responses, we analyzed the production of IL-10 by splenic and peritoneal B cells *ex vivo*. Albeit the level of IL-10 production in B cells of unchallenged APRIL-Tg and control mice were not significantly different (Figure 8E), the percentages of IL-10 producing peritoneal and splenic B cells were significantly higher in APRIL-Tg as compared to littermates 3 days after oxazolone challenge (Figures 8F,G). A similar increase in IL-10 producing peritoneal and splenic B cells was seen during CIA in APRIL-Tg vs. wildtype littermate animals (data not shown). In order to demonstrate that the APRIL-induced IL-10-producing peritoneal B cells are not only associated with but responsible for the dampening of the disease in APRIL-Tg animals, we depleted the peritoneal B1 cells (containing the IL-10 producing population) by hypotonic shock with repeated intraperitoneal injections of distilled water. Distilled water-treated APRIL-Tg mice developed EAE with a disease severity comparable to that of WT mice (Figure 8H), indicating that a decrease in IL-10-producing peritoneal B cells abolishes the disease dampening effects of ectopic APRIL expression. Collectively, these data indicate that APRIL can induce during the course of disease a population of IL-10-producing regulatory B cells which dampens disease severity *in vivo*. It has already been described that the levels of regulatory IL-10-producing B cells are inversely correlated to the disease activity score in 28 joints in RA patients (53). Therefore, and based on our human *in vitro* data and murine *in vivo* data, we finally assessed a possible correlation between APRIL and IL-10 in the joints of patients with inflammatory arthritis. A highly significant correlation between APRIL and IL-10 levels is observed in the synovial fluid of inflammatory arthritis patients (Figure 8I), suggesting that APRIL modulates IL-10 production not only in mice but also in humans.

## Discussion

This study identified a subset of human IL-10 producing B cells within the IgA switched B cell population. IL-10 production is considered the hallmark of Bregs and has previously been reported to be present amongst subsets of CD19^+^CD24^hi^CD38^hi^CD1d^+^ transitional B cells (6), CD24^hi^CD27^+^ B cells (16), and CD24^−^CD27^hi^CD38^hi^ plasmablasts (7) in humans. Here we identified a distinct subset of human Bregs that does not express most of the above mentioned phenotypical markers, but is defined by the expression of IgA. However, it has not been described if the other human Bregs were confined to a specific isotype, in particular IgA. Moreover, in contrast to previously described subsets, the cells characterized here did not require additional activation, such as stimulation with CpG or PMA/IO, to produce significant amounts of IL-10. Whereas, these data indicate clear differences between previously described human Bregs and the IgA^+^ IL-10 producing B cells, it remains impossible to assess whether IL-10 production is really cell-intrinsic as our experiments require continuous exposure to CD40L and IL-21 to keep the cells alive *in vitro*.

The fact that phenotypically distinct human B cell subsets can produce IL-10 in the presence of the appropriate stimuli raises the question whether Bregs represent a “reactive” functional population of B cells rather than a programmed, specifically differentiated population of cells. Signals required for the differentiation of previously described human Bregs remain poorly understood. IFN-α produced by TLR9-activated plasmacytoid DCs promoted the expansion of IL-10 producing CD24^+^CD38^hi^ Bregs (54). Here, we provide evidence for a distinct differentiation program for the IgA^+^ IL-10-producing B cell subset as (1) not all IgA^+^ B cells were able to produce IL-10 *ex vivo*, (2) only APRIL-induced, and not TGF-β or BAFF-induced, IgA^+^ B cells were IL-10 positive *in vitro*, (3) APRIL stimulation of already isotype switched IgA memory B cells was unable to augment IL-10 production, and (4) IL-10 production was, albeit maintained or augmented by TLR ligands, not critically dependent on additional activation. Besides APRIL, TGF-β and IL-21 have been described as factors that induces class switching of human B cells to IgA (40, 41). Whereas, we did not observe significant IgA class switching with the single cytokines, we confirmed that IL-21 combined with TGF-β allows human naïve B cells to class switch toward IgA. However, in contrast to APRIL, class switching to IgA induced by TGF-β did not result in B cells with regulatory properties. Interestingly, a recent publication using a mouse model of prostate cancer has identified regulatory IgA^+^ plasma cells that suppressed tumor-directed cytotoxic T cell functions via the expression of IL-10 and PD-L1 (14). TGF-β signaling and IgA CSR were required for the development of this murine Breg subset (14). Although TGF-β induced IgA CSR in human B cells, signaling via TGF-β did not lead to IL-10 and PD-L1 expression and induction of immunoregulatory properties. This discrepancy might be explained either by the difference between human and murine Bregs, or by the fact that our human TGF-β-induced IgA^+^ B cells are cultured *in vitro* under precisely known conditions, whereas the murine IgA^+^ Bregs were induced in a complex *in vivo* environment containing other immunoregulatory molecules besides TGF-β. Another study performed in mice has shown that BAFF induces IL-10-producing Bregs with a CD1d^hi^CD5^+^ phenotype (55). In our cultures, BAFF was not able to induce significant amounts of IL-10 producing B cells. Again, this discrepancy might be explained by differences between human and murine Bregs. Another explanation could be the different cell of origin used in the experiments, here we start with naïve B cells isolated from peripheral blood, whereas the Yang et al. study uses splenic B cells.

Mechanistically, we could confirm that by RNA sequencing that the transcriptional program of APRIL-induced IgA^+^ cells is fundamentally different from TGF-β-induced IgA^+^ cells, and that addition of TGF-β together with APRIL overcomes the APRIL-induced molecular profile. Blocking experiments also revealed that the APRIL-induced capacity to produce IL-10 is dependent on TACI signaling. An intriguing finding is that, albeit BAFF induced only a very low level of IgA isotype switching and no IL-10 secretion by these rare IgA^+^ cells, the transcriptional profile of BAFF-induced IgA^+^ B cells was indistinguishable from that of APRIL-induced IgA^+^ cells. Further research should clarify if BAFF-R signaling, which is unique to BAFF, might control the expression of immunoregulatory molecules at the translational level and whether BAFF signaling through TACI in the absence of BAFF-R activation may upregulate IL-10 production.

Human regulatory B cells have been described to control inflammatory responses via the inhibition of Th1 and Th17 differentiation, suppression of CD8^+^ T cell effector functions, and the conversion of CD^4+^ T cells into Tregs and Tr1 cells (6, 56–58). Additionally, CD24^hi^CD27^+^ Bregs can suppress TNF-α production by monocytes via IL-10 (16). Although we could not detect a clear impact of the human APRIL-induced IgA^+^ Bregs on T cell polarization, these Bregs significantly suppressed CD4^+^ T cell proliferation and TNF production and induced FoxP3^+^ Tregs in an IL-10 dependent fashion. APRIL-induced IgA^+^ Bregs also suppressed TNF production by macrophages through both IL-10 and PD-L1. Collectively, these data indicate that this human Breg subset express an array of immunoregulatory molecules that functionally contribute to suppression of T cell and myeloid responses *in vitro*, which is consistent with the profile of other human Breg subsets.

These *in vitro* findings raised the question whether the APRIL-induced IgA^+^ B cells also exert immunoregulatory functions *in vivo*. Here, we demonstrated that APRIL tg mice are protected from autoantibody-independent inflammatory disease models, that this was associated with an increase in IL-10 producing B cells in spleen and peritoneum, and that depletion of peritoneal cells abrogated the protection conferred by APRIL overexpression. Albeit this data further supports the concept of immunoregulation by APRIL *in vivo*, the models used here have a number of intrinsic limitations. First, it remains to be determined of the IL-10 production observed during inflammation in the APRIL tg mice is restricted to IgA^+^ B cells. Second, the peritoneal depletion experiments may have targeted other regulatory cell populations besides the IL-10 producing B cells. Thirdly, it remains unclear if APRIL overexpression exerts its function only through IL-10 or also by other immunoregulatory molecules in this model. And finally, the relative contribution of APRIL-induced Bregs vs. other Bregs remains to be determined in more physiological conditions.

Whereas, addressing these questions in complex animal models, including APRIL tg x B cell-specific IL-10 KO mice, is of obvious scientific interest, the major limitation remains the clear differences between murine and human Bregs. Accordingly, the major question raised by the data presented here is the presence, role, and relevance of APRIL-induced IgA^+^ Bregs in human immunopathology. We demonstrated that IL-10 is indeed produced by a subset of peripheral blood IgA^+^ memory B cells *ex vivo*. Additionally, we have previously reported a direct effect of APRIL, but not BAFF, on IL-10 production by CpG activated human B cells derived from not only healthy donors but also RA patients (27). Finally, we showed a strong correlation between APRIL and IL-10 in the inflamed synovial tissue. Collectively, these observations indicate the potential relevance of this cell population for immune homeostasis and immunopathology in humans and suggest that selective targeting of BAFF (or BAFF-R) rather than blocking both BAFF and APRIL-induced pathways may offer potential advantages. The difference in BAFF and APRIL with regard to the induction of a regulatory profile in B cells might contribute to explain why atacicept, a TACI-Fc fusion protein inhibiting both BAFF and APRIL signaling, did not reach the primary end point in a phase II trial in RA (59), whereas belimumab, which specifically antagonizes BAFF, is approved for the treatment of SLE and obtained significant efficacy on ACR20 criteria in RA (24, 25, 60).

## Ethics Statement

The studies involving human subjects were carried out in accordance with the recommendations of the medical ethical committee of the AMC Amsterdam, The Netherlands with written informed consent from all subjects. All subjects gave written informed consent in accordance with the Declaration of Helsinki. The protocol was approved by the medical ethical committee of the AMC Amsterdam, The Netherlands.

The studies involving animal subjects were carried out in accordance with national and institutional guidelines. Experiments were authorized by the respective French authorities (Départementale des service vétérinaires de la Prefecture de l'Herault), Permit number D 34-172-16.

## Author Contributions

CF, MH, and DB were involved in the study concept and design. CF, NvU, NY, GF, LvD, and LF performed the experiments. MH provided the APRILtg mice. The retroviral vectors containing BCL-6 and BCL-xL have been generated by AIMM therapeutics. CF and DB analyzed and interpret the data. All authors were involved in drafting the article or revising it critically for important intellectual content and approved the final version.

### Conflict of Interest Statement

HS owns stock or stock options in AIMM Therapeutics and is Chief Science Officer of AIMM Therapeutics. DB is an employee of UCB and received consultancy fees/grants from AbbVie, Pfizer, MSD, Roche, BMS, Novartis, Eli Lilly, Janssen, Glenmark, Boehringer-Ingelheim. The remaining authors declare that the research was conducted in the absence of any commercial or financial relationships that could be construed as a potential conflict of interest.
