# Supplementary material for: APRIL Induces a Novel Subset of IgA+ Regulatory B Cells That Suppress Inflammation via Expression of IL-10 and PD-L1
Source: Front Immunol. 2019 Jun 14;10:1368. doi: 10.3389/fimmu.2019.01368 (PMC6587076; doi:10.3389/fimmu.2019.01368)
Supplement: Supplementary file 3 [file Data_Sheet_1.PDF]

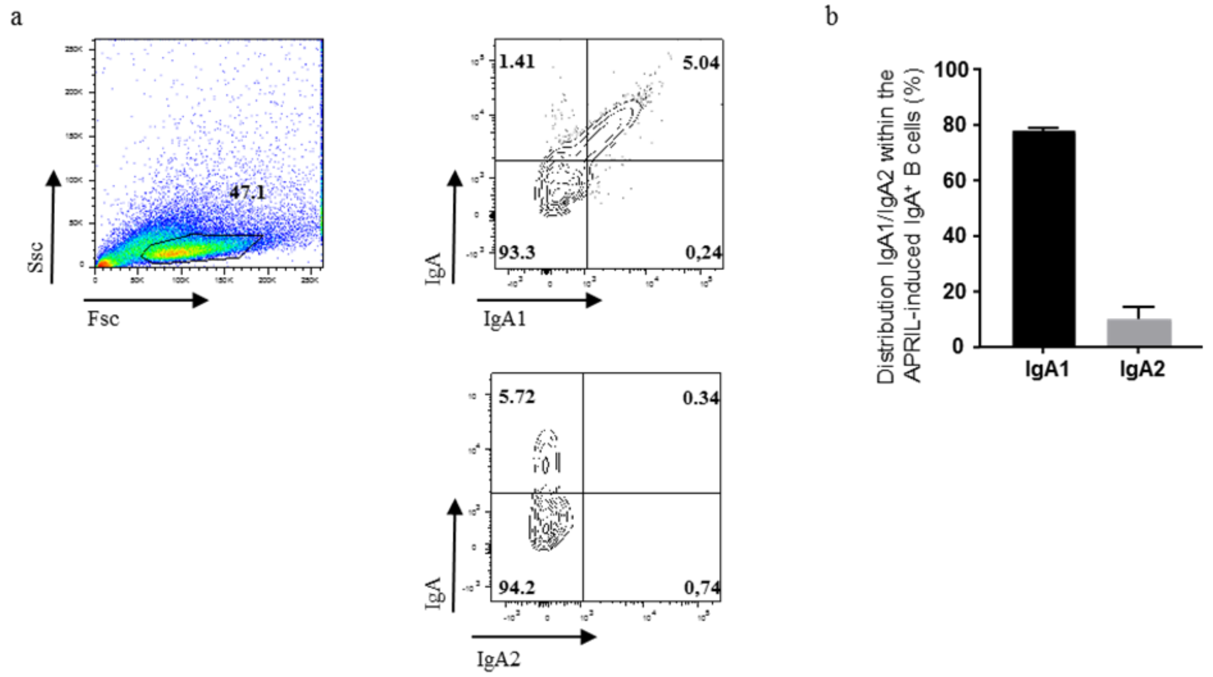

Supplementary Figure 1. APRIL-induced IgA^{+} B cells belong predominantly to the IgA1 isotype. (A-B) Human naïve B cells were cultured for 6 days in the presence of CD40L-expressing fibroblasts, IL-21 and APRIL, where after the cells were harvested and the expression of IgA, IgA1 and IgA2 was assessed using flow cytometry. (A) Representative dot plots are shown. N=4. (B) Percentages of IgA1 and IgA2 of total APRIL-induced IgA are shown. Mean ± SD is shown, n=4.

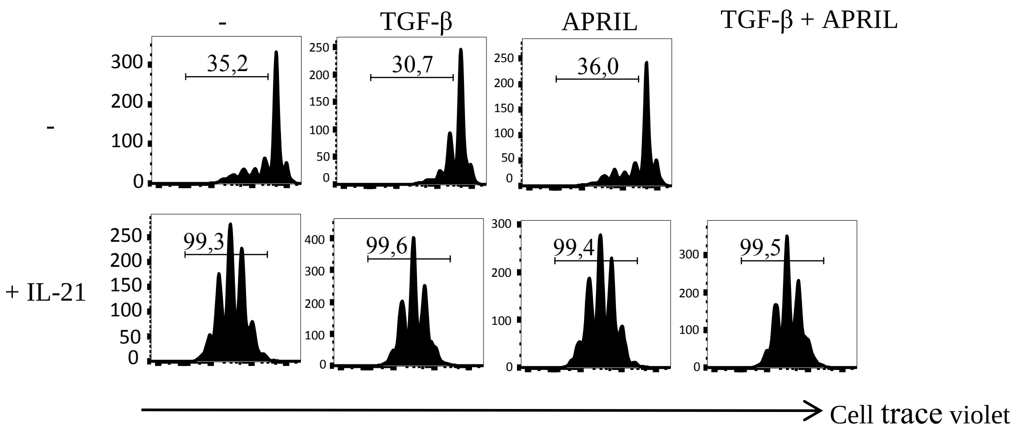

Supplementary Figure 2. Human B cells require CD40 ligand stimulation and IL-21 to proliferate i*n vitro*. Human naïve B cells were isolated from peripheral blood, labelled with cell trace violet and cultured for 6 days in the presence of CD40L-expressing fibroblasts and indicated factors. After 6 days, the cells were harvested and proliferation was assessed using flow cytometry. Representative histograms are shown (n = 3).

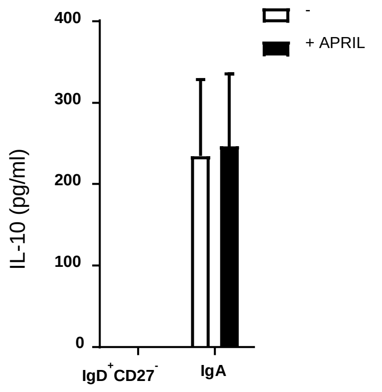

Supplementary Figure 3. ***In vivo*** **class switched IgA^+ B cells do not increase their IL-10 production upon stimulation with APRIL.** Peripheral blood-derived naïve ($CD19^+IgD^+CD27^-$) and $CD19^+IgA^+$ B cells were sorted and stimulated for 24h +/- APRIL, where after the production of IL-10 was analyzed in the supernatants using ELISA. Mean ± SD are shown, n=3.

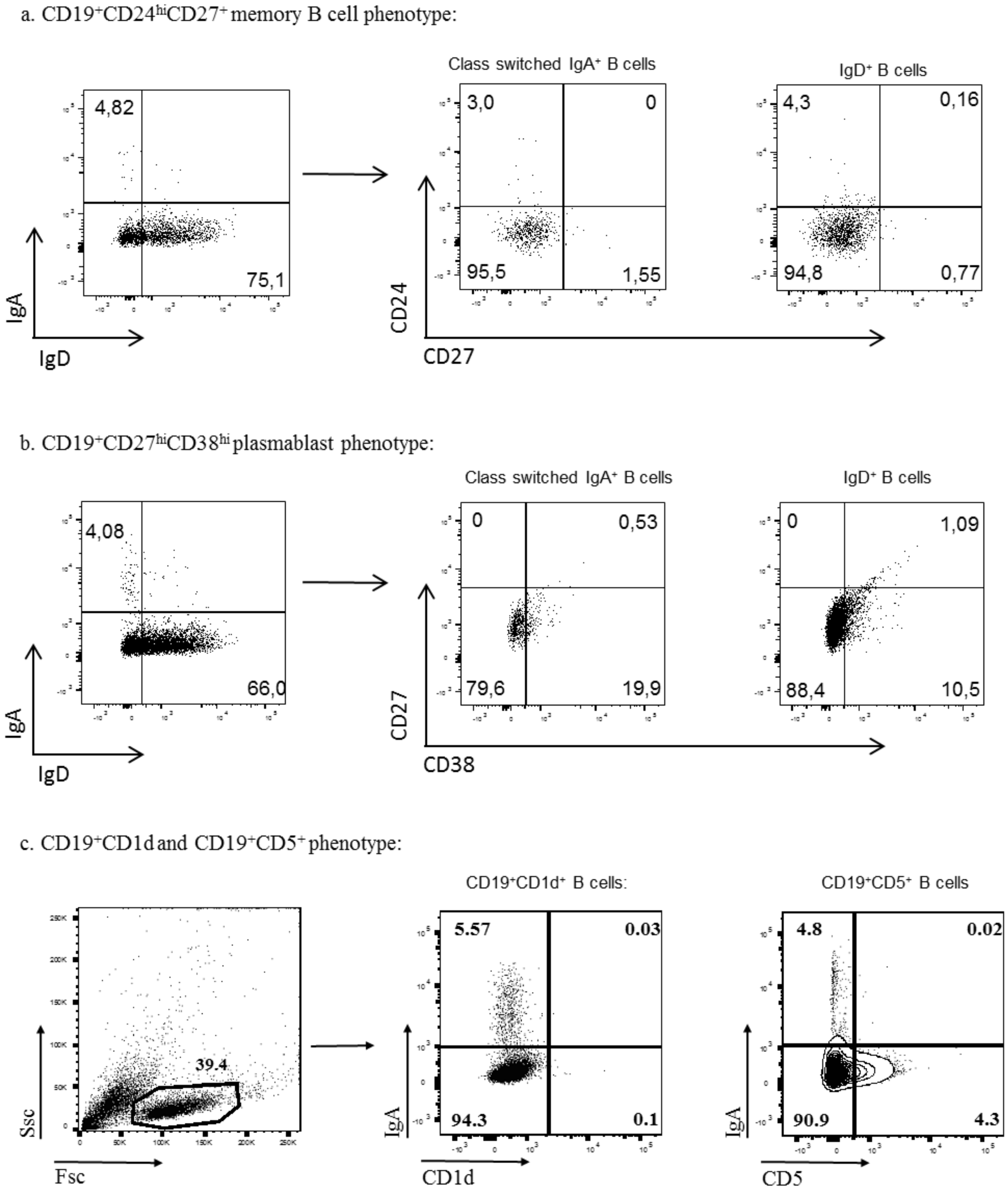

Supplementary Figure 4. APRIL-induced IgA$^+$ B cells do not obtain the phenotypical markers of CD19$^+$CD24^{hi}CD27$^+$ memory B cells or plasmablasts, nor do they acquire expression of CD1d or CD5. Human naïve B cells were cultured for 6 days in the presence of CD40L-expressing fibroblasts, IL-21 and APRIL, where after the cells were harvested and the expression of phenotypical markers was assessed using flow cytometry. Representative dot plots are shown. N=3.

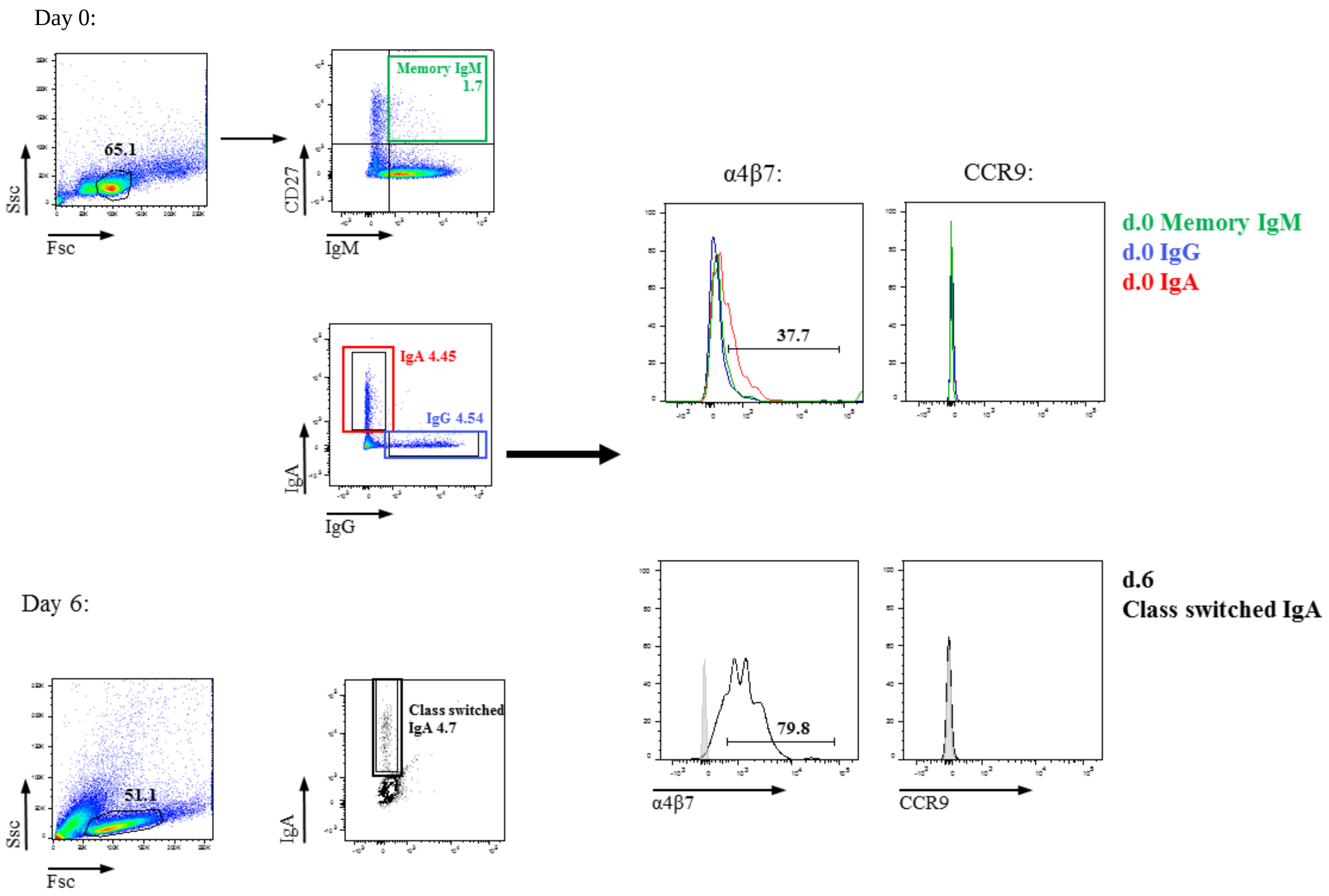

Supplementary Figure 5. APRIL-induced IgA^{+} B cells upregulate the gut-homing receptor α4β7. Expression of α4β7 and CCR9 was analysed on peripheral blood-derived memory CD27^{+}IgM^{+}, IgG^{+} and IgA^{+} B cells directly after isolation (day 0). Next, naïve B cells were sorted and cultured for 6 days in the presence of CD40L-expressing fibroblasts, IL-21 and APRIL, where after the cells were harvested and the expression of α4β7 and CCR9 was analysed on APRIL-induced IgA^{+} B cells using flow cytometry. Representative dot plots are shown. N=4.

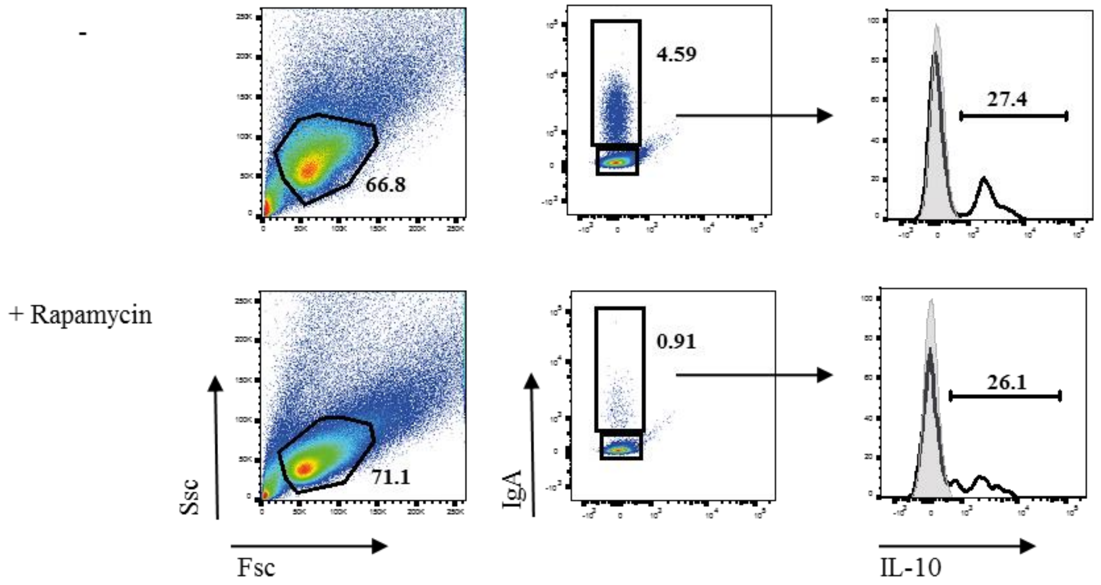

Supplementary Figure 6. APRIL induces CSR to IgA, but not IL-10 production, via the TACI-mediated mTOR pathway. Human naïve B cells were cultured for 6 days in the presence of CD40L-expressing fibroblasts, IL-21 and APRIL. 10 nM rapamycin was added on day 0 of the cultures. After 6 days the cells were harvested and the expression of IgA and IL-10 was assessed using flow cytometry. Representative dot plots and histograms are shown. N=4.
